# Engineered IgA-Fc fusion protein with bioactive nanobody neutralizes SARS-CoV-2 variants with mucosal delivery potential

**DOI:** 10.1016/j.jbc.2026.111210

**Published:** 2026-01-28

**Authors:** Rachel M. Golonka, Lauren E. Intravaia, Ala M. Shaqra, Qi Li, Yongzhi Chen, Fiachra Humphries, Nese Kurt-Yilmaz, Kate A. Fitzgerald, Celia A. Schiffer, Yang Wang, Lisa A. Cavacini

**Affiliations:** 1 Division of Innate Immunity, Department of Medicine, University of Massachusetts Chan Medical School, Worcester, Massachusetts, USA; 2 Department of Biochemistry and Molecular Biotechnology, University of Massachusetts Chan Medical School, Worcester, Massachusetts, USA

**Keywords:** antibody engineering, drug development and delivery, Fc-fusion protein, immunogenicity, immunoglobulin A (IgA), monoclonal antibodies, recombinant protein expression, SARS-CoV-2, single-domain antibody (sdAb, nanobody), structural modeling

## Abstract

The COVID-19 pandemic accelerated the development of monoclonal antibodies (mAb) targeting SARS-CoV-2, with IgG1-based mAbs dominating the therapeutic landscape. However, IgA—the predominant immunoglobulin at mucosal surfaces—represents a promising alternative for respiratory infections due to its natural role in immune exclusion and pathogen neutralization. Here, we engineered IgA-Fc fusion proteins conjugated with nanobodies (V_H_H-IgA) derived from immunized llamas to neutralize SARS-CoV-2 variants. Phage display libraries generated from Delta and Omicron receptor-binding domain (RBD) immunized llamas yielded 248 unique V_H_H sequences, with fifty candidates selected based on binding reactivity and neutralization potency. V_H_H-IgA fusion proteins were expressed in Expi293 cells, and top candidates exhibited high binding affinities (EC_50_ < 0.2 nM) and potent neutralization (IC_50_ < 40 pM) against multiple SARS-CoV-2 variants, including Omicron Ba.1 and XBB. Structural modeling predicted that the leading V_H_H-IgA candidates 2D4, 1C2, and 2D10 adopt distinct binding conformations to accommodate amino acid sequence variations on the Omicron RBD domain. *In vitro* assays demonstrated that 2D4, 1C2, and 2D10 neutralized authentic Omicron variants of concern, with 2D4 exhibiting the broadest activity. *In vivo*, intranasal administration of 2D4 V_H_H-IgA significantly reduced SARS-CoV-2 XBB viral loads in the lungs of infected K18-hACE2 mice. These findings highlight the therapeutic potential of IgA-based nanobody fusion proteins as mucosal antivirals against SARS-CoV-2. Our work positions V_H_H-IgA fusion proteins as a platform for developing next-generation biologics to combat respiratory pathogens at mucosal surfaces.

Monoclonal antibodies (mAb) have revolutionized the treatment of various diseases, particularly cancer, autoimmune disorders, and viral infections. Their development and use in viral infections have been evolving for decades, with milestones marking key advances in science and technology. More recently, the COVID-19 pandemic sparked rapid development and usage of mAbs, such as bamlanivimab, casirivimab, and imdevimab, to target the spike protein of the SARS-CoV-2 virus (1). These mAb, along with all other current clinically used therapeutic antibodies, have a human IgG1 blueprint (2, 3, 4). Human IgG1 is the main backbone for generating mAbs because its pharmacokinetics, Fc receptor engagement, manufacturing, and safety are well established (5). While human IgG1 mAbs are essential for treating systemic diseases, the design of mAbs to target mucosal diseases, like gastrointestinal or respiratory tract infections, might benefit from a different immunoglobulin blueprint to better enhance immune responses.

The immunoglobulin IgA makes up a small proportion of serum antibodies and functions as an immunoregulatory protein in circulation because blood is typically a microbial-free environment. In contrast, IgA is the predominant immunoglobulin at mucosal surfaces and accounts for a significant portion (∼70%) of daily immunoglobulin production in mammals (6). IgA is widely recognized for maintaining gut microbiota homeostasis, preventing pathogens from attaching to mucosal epithelial cells (*i.e.,* immune exclusion), and promoting inflammatory defense responses, to name a few functions (7, 8, 9). These functions of IgA have made it an important clinical asset in diagnosing the stage/severity of infections and in determining vaccination efficacy, as recently seen in patients infected with or vaccinated against SARS-CoV-2 (10, 11, 12). Several recent studies of SARS-CoV-2 infection demonstrated that IgA has a greater role in the neutralizing antibody response than IgG, and that IgA efficacy increases when in its dimeric or secretory form (13, 14, 15). Altogether, this supporting evidence suggests that designing mAbs with an IgA blueprint could be valuable for treating mucosal diseases.

There have been some advancements in discovering, designing, and characterizing human IgA mAbs in the context of SARS-CoV-2 (16, 17), HIV (18, 19), influenza (20, 21), and measles (22). Although IgA can be produced recombinantly and maintain effector function, it is more challenging to manufacture in large quantities due to its structure and the fact that it is typically secreted as a dimer (with a secretory component) in mucosal tissues. An option to circumvent this limitation is to generate fusion proteins that contain the immunoglobulin Fc domain attached to a bioactive moiety, such as viral neutralizing peptides, antigens, or single-domain antibodies (*alias* nanobody). Nanobodies are small (∼15 kDa size) antibody fragments found in the Camelidae family, including camels and llamas, as well as in cartilaginous fish. They are also known as heavy-chain-only antibodies (HCAb or V_H_H) because they lack light chains and CH1 domains (23). Compared to viral peptides and antigens, nanobodies are a superior Fc-fusion partner due to their enhanced target specificity and affinity, improved stability and pharmacokinetics, smaller size, better tissue penetration, and cost-effectiveness to produce. Following the same trend as mAbs, though, Fc-fusion proteins have predominantly used an IgG backbone [as reviewed (24, 25)], and only a few studies have investigated IgA-Fc fusion proteins (26, 27, 28).

In this study, we designed and characterized nanobody IgA-Fc fusion proteins (V_H_H-IgA) using SARS-CoV-2 as a viral infection model. Our findings demonstrated that V_H_H-IgA are effective at neutralizing SARS-CoV-2 *in vitro* and *in vivo*, with an emphasis on broadly protective V_H_H-IgA against Omicron and its subvariants. Structural predictions illustrate that V_H_H-IgA proteins have distinct conformations to the highly mutated Omicron variant that may contribute to their efficacy. Collectively, our work demonstrates V_H_H-IgA fusion proteins as a next-generation biologic to combat SARS-CoV-2 respiratory infection.

## Results

### Discovery of anti-RBD V_H_H from immunized llama and phage display library

**Figure 1:**
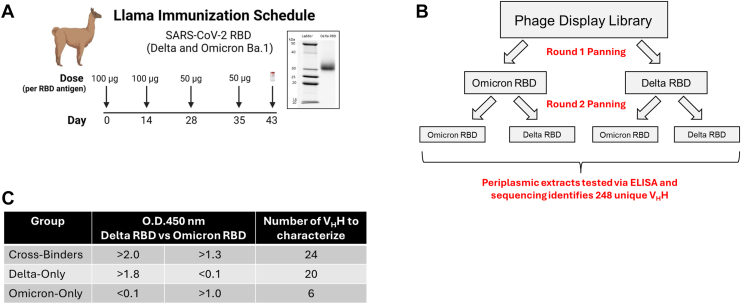
**Llama immunization schedule, phage display library, and ELISA thresholds.***A*, schematic diagram of the llama immunization with Delta and Omicron Ba.1 RBD. *B*, illustration of the two-panning method for phage display library. *C*, table that states O.D. 450 nm thresholds to sort leading candidates for recombinant expression.

To generate nanobodies (*i.e.,* V_H_H) with broad cross-reactivity against SARS-CoV-2, two llamas were subcutaneously immunized with a mixture of the RBD proteins from the Omicron (B.1.1.529, *alias* Ba.1) and Delta (B.1.1617.2) variants (Fig. 1*A*). Serum response to the RBD antigens was measured by ELISA, and peripheral blood mononuclear cells were isolated to generate two phage-display libraries as previously described (29). Libraries were initially used for panning immobilized Omicron Ba.1 or Delta RBD, and outputs from these assays were used for subsequent auto- and cross-panning (Omicron-Omicron, Omicron-Delta, Delta-Delta, Delta-Omicron) (Fig. 1*B*). Periplasmic extracts were prepared, assessed for binding to both RBD antigens *via* ELISA, and the 288 identified cross-variant and single-variant binders were sent for sequence determination (Fig. 1*B*). Alignment analysis removed forty repeats, leaving 248 unique nanobody sequences. Upon applying O.D. 450 nm thresholds on the ELISA results (Fig. 1*C*), we selected 24 V_H_H cross-binders, 20 V_H_H Delta-only binders, and 6 V_H_H Omicron-only binders for further characterization.

### Selection of V_H_H-IgA with high binding affinity to RBD and potent neutralization against SARS-CoV-2 variants

The small size of the V_H_H structure often results in rapid clearance after intravenous administration. One strategy to address this challenge is fusing the V_H_H to the Fc region of immunoglobulin, which also enables mucosal administration as a mode of drug delivery (30, 31). In the context of SARS-CoV-2 infection, IgG has been the predominant Fc backbone to make V_H_H fusion proteins (32). Notably, our previous research in parallel with others demonstrated the ability of monoclonal IgA to neutralize SARS-CoV-2—even outperforming monoclonal IgG in some cases (13, 15, 16, 17). As such, we engineered the top 50 anti-RBD V_H_H as fusion proteins with the Fc domain of IgA1 (termed V_H_H-IgA) as previously described (27, 33).

**Figure 2:**
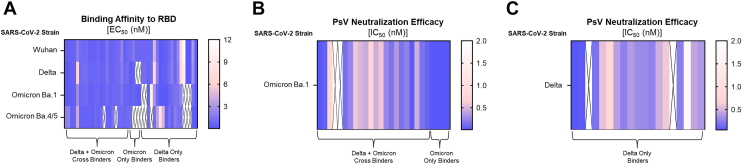
**Top 50 V_H_H-IgA candidates screened for RBD reactivity and neutralization efficacy.***A*, heat map depicting the EC_50_ (nM) of each V_H_H-IgA against the ancestral strain and Delta, Omicron Ba.1, and Omicron Ba.four-fifths RBD from ELISA. *B*, heat map depicting the IC_50_ (nM) of V_H_H-IgA in the cross-binder and Omicron-only binder groups against the Omicron Ba.1 pseudovirus. *C*, heat map depicting the IC_50_ (nM) of V_H_H-IgA in the Delta-only binders’ group against the Delta pseudovirus. n = 22 for cross-binders, n = 4 for Omicron-only binders, and n = 19 for Delta-only binders. There were five recombinant proteins excluded from data presentation because they had no binding affinity or neutralization activity. Data is plotted based on the average ± SEM from at least 3 independent experiments. The ‘X’ stands for non-binding.

V_H_H-IgA were expressed in Expi293 cells, and the purified fusion proteins were first assessed for binding affinity to the RBD antigens from the ancestral strain and Delta, Omicron Ba.1, and Omicron Ba.four-fifths variants (Fig. 2*A*). Out of the three V_H_H-IgA groups, the cross-binders had the broadest binding affinity with an average effective concentration (EC_50_) of 0.9 nM against the four strains. Omicron-only binders retained strong binding affinity toward the expected Omicron Ba.1 variant (average EC_50_ of 0.5 nM) with little to no cross-reactivity to the ancestral strain and unexpectedly Omicron Ba.four-fifths RBD. Yet, we observed that two of the Omicron-only binders (group name designated during the phage-display stage) had binding affinity to Delta RBD when expressed recombinantly. Similarly, while V_H_H-IgA from the Delta-only group (group name designated during the phage-display stage) bound decently to RBD from the ancestral strain (average EC_50_ of 2.0 nM) and Delta (average EC_50_ of 2.1 nM), 70% (14/20) of the fusion proteins also bound to Omicron Ba.1 and/or Ba.four-fifths RBD. Despite the emergence of cross-reactivity in the Omicron-only and Delta-only groups, we retained the original group labels for simplicity.

We next performed a lentiviral-based pseudovirus (PsV) assay on hACE2-transfected 293T cells and determined the neutralization efficacy of each V_H_H-IgA against its predominant SARS-CoV-2 variant. Impressively, the Omicron-only binders had potent efficacy against the Omicron Ba.1 PsV with an average inhibitory concentration (IC_50_) of 13.5 pM (Fig. 2*B*). In comparison, the V_H_H-IgA cross-binders (Fig. 2*B*) and Delta-only binders (Fig. 2*C*) had a broader range of neutralization against Omicron Ba.1 PsV (IC_50_: 16.5–730 pM) and Delta PsV (IC_50_: 55.3–1,790 pM), respectively. Moreover, there were a couple of cross-binders and Delta-only binders that did not exhibit neutralization activity despite having binding affinity toward RBD. Of note, there were five V_H_H-IgA that had no affinity to any RBD antigens nor PsV neutralization activity despite being expressed similarly to the other candidates. These V_H_H-IgA (two cross-binders, two Omicron-only binders, and one Delta-only binder) were excluded from additional screenings. The V_H_H-IgA (n = 10) that exhibited potent PsV neutralization activity (IC_50_ < 100 pM) were selected for another round of characterization.

**Figure 3:**
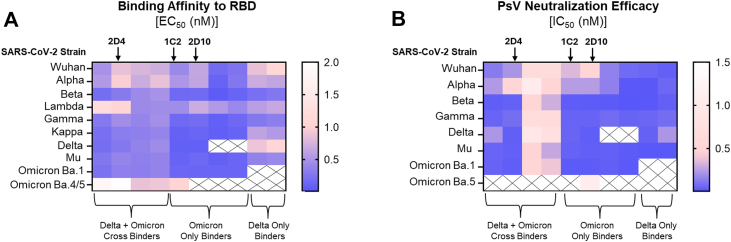
**Top 10 V_H_H-IgA candidates tested for RBD affinity and neutralization efficacy against SARS-CoV-2 VOC.***A*, heat map depicting the EC_50_ (nM) of each V_H_H-IgA against the ancestral strain Alpha, Beta, Lambda, Gamma, Kappa, Delta, Mu, Omicron Ba.1, and Omicron Ba.four-fifths RBD from ELISA. *B*, heat map depicting the IC_50_ (nM) of each V_H_H-IgA against the ancestral strain, Alpha, Beta, Gamma, Delta, Mu, Omicron Ba.1, and Omicron Ba.5 pseudovirus. n = 4 for cross-binders, n = 4 for Omicron-only binders, and n = 2 for Delta-only binders. Data are plotted based on the average ± SEM from at least 3 independent experiments. The ‘X’ stands for non-binding.

A total of four cross-binders, four Omicron-only binders, and two Delta-only binders from the initial screen were further tested for binding affinity against RBD from 10 SARS-CoV-2 variants (Fig. 3*A*). Out of the three groups, V_H_H-IgA from Omicron-only binders had noteworthy binding affinities from the Gamma to Omicron Ba.1 variants (average EC_50_ of 0.1 nM). Comparatively, Delta-only binders were weaker (average EC_50_ of 0.6 nM) or absent binding affinities to all tested RBD antigens. The majority of cross-binders also had moderate to weak binding affinities, but there were a couple of V_H_H-IgA that exhibited stronger reactivity to the Gamma and Omicron Ba.1 variants. Of note, only five of the 10 V_H_H-IgA from all three groups had antigen reactivity to Omicron Ba.four-fifths RBD.

We next performed another PsV screening on the V_H_H-IgA, but we expanded to evaluate their neutralization efficacy against eight SARS-CoV-2 variants (Fig. S2). Despite observing lower binding affinity of several cross-binders and Delta-only binders *via* ELISA, we observed that the reactivity is sufficient to potently neutralize nearly all SARS-CoV-2 variants (average IC_50_: 85.5 pM). However, two V_H_H-IgA cross-binders had the opposite result with moderate binding affinity but weak PsV neutralization activity (IC_50_ > 300 pM). Omicron-only binders were found to be dominant in neutralizing PsV from Beta to Omicron Ba.1 strains (average IC_50_: 30.8 pM), which correlated with their binding affinity data. Interestingly, only one V_H_H-IgA (from the Omicron-only group) neutralized Omicron Ba.four-fifths, *albeit* weakly. As current sub-variants of SARS-CoV-2 are derived from the Omicron lineage, we selected our top fusion protein candidates as those with strong PsV neutralization activity (IC_50_ < 40 pM) against Omicron Ba.1 and those that exhibited cross-reactivity to all tested strains (besides Omicron Ba.four-fifths). Accordingly, we identified three V_H_H-IgA candidates, one original cross-binder named 2D4 and two original Omicron-only binders named 1C2 and 2D10, to move forward in evaluating their therapeutic potential.

### Structural modeling of lead nanobodies binding to RBD

**Figure 4:**
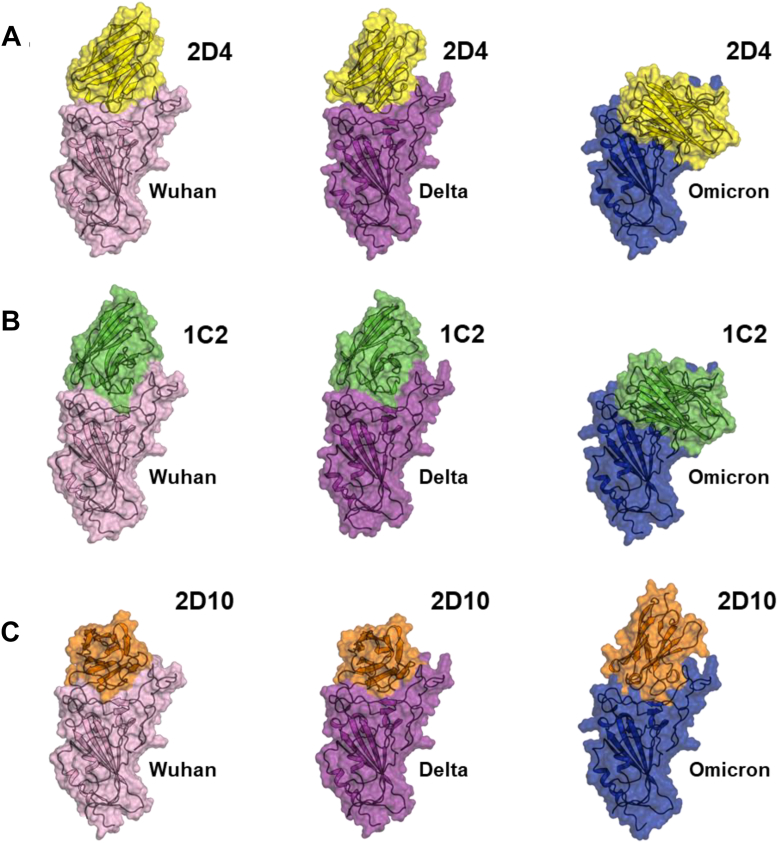
**AlphaFold2 multimer predicted structure complexes between the nanobody and the proteins.** Cartoon and surface representation of the highest confidence AlphaFold2 predictions of nanobodies (*A*) 2D4 (*yellow*), (B) 1C2 (*green*), and (*C*) 2D10 (*orange*) in complex with RBD variants WA (*pink*), Delta (*purple*), and Omicron (*blue*), showing multiple nanobody binding modes depending on the RBD sequence.

To investigate the binding mode of leading nanobodies, AlphaFold2-Multimer was used to generate structural models of nine nanobody-RBD complexes from the amino acid sequences of nanobodies 2D4, 1C2, and 2D10 (Table S1) and RBD ancestral strain and variants Delta, and Omicron Ba.1 (Fig. 4, *A*–*C*). Analysis of the predicted structures revealed that all three leading nanobodies can adopt multiple binding modes to accommodate amino acid differences across RBD variants. Note that ancestral strain and Delta differ at only two amino acid residues, whereas ancestral strain and Delta differ from Omicron Ba.1 at 14 and 15 residues, respectively. Each of the leading nanobodies displays a common pattern in which they bind RBD variants *via* an extensive surface interaction involving the complementarity-determining region (CDR) loops. While all three candidates had a similar conformation toward ancestral strain and Delta, they have a distinct conformation to the Omicron Ba.1 variant, which is likely due to the highly mutated RBD surface of this variant. Taken together, the predicted structural models of the nanobody-RBD complexes reveal a potential mechanism by which the leading nanobodies exhibit broad cross-reactivity against SARS-CoV-2 variants.

### Leading V_H_H-IgA candidates neutralize authentic Omicron variants of concern *in vitro* and *in vivo*

**Figure 5:**
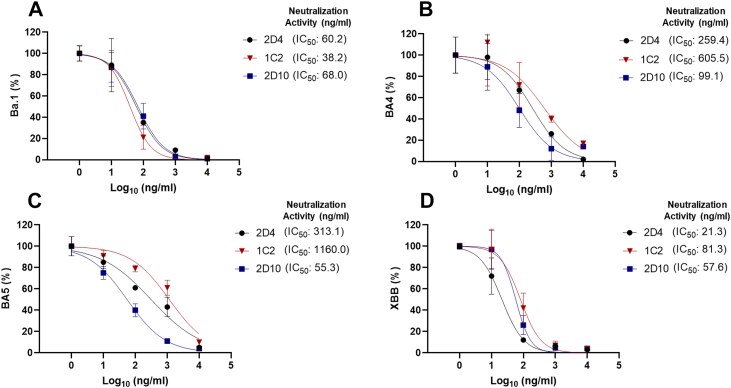
**Top 3 V_H_H-IgA candidates potently neutralize authentic SARS-CoV-2 Omicron VOC *in vitro*.** Vero E6 cells were pretreated with various concentrations of V_H_H-IgA for 2 h before infection with SARS-CoV-2 Omicron VOCs (*A*) Ba.1, (*B*) Ba.4, (*C*) Ba.5, and (*D*) XBB at an MOI=0.05. After 24 h of infection, the RNA was extracted from the cells and then reverse-transcribed for qPCR analysis. Gene expression was normalized to the *Gapdh* housekeeping gene, and data are presented as a % of neutralization with respect to non-treated infected cells. Data are plotted based on the average ± SEM from at least 3 independent experiments.

To explore the potential of 2D4, 1C2, and 2D10 V_H_H-IgA as a prophylactic candidate, we determined if the nanobody fusion protein would neutralize live authentic Omicron VOCs using VeroE6 cells. The neutralization efficacy of the three leading candidates against the SARS-CoV-2 authentic virus was tested by measuring viral RNA levels. Out of all the variants tested, 2D4, 1C2, and 2D10 had the most potent neutralization activity (IC_50_ values < 100 ng/ml) against Omicron Ba.1 and XBB (Fig. 5, *A* and *D*). Comparatively, the neutralization efficacy of each V_H_H-IgA was significantly reduced against Ba.4 (Fig. 5*B*) and Ba.5 (Fig. 5*C*) variants. Yet, it is noteworthy that 2D10 was the only one with moderate neutralization activity against both Ba.four-fifths variants, which matched the ELISA and PsV results during the second round of screening. These data collectively demonstrate that all three leading V_H_H-IgA had high efficacy to neutralize authentic SARS-CoV-2.

**Figure 6:**
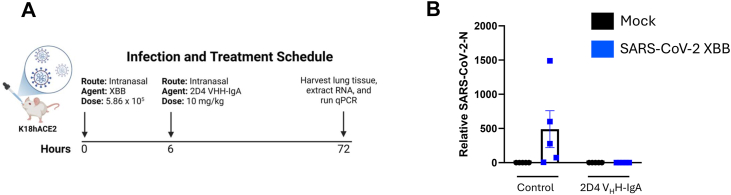
**2D4 V_H_H-IgA potently neutralizes authentic SARS-CoV-2 XBB *in vivo*.***A*, schematic diagram of the post-exposure treatment model in K18hACE2 mice. After 72 h of infection, RNA was extracted from the lung tissue and then reverse-transcribed. *B*, Dot plot depicting the relative value of viral RNA represented by qPCR analysis of SARS-CoV-2-N. Gene expression was normalized to the TATA-binding protein (*Tbp*) housekeeping gene. n = 5 mice/group. Data is plotted based on the average ± SEM.

At the start of these experiments, the main SARS-CoV-2 variant of concern (VOC) was the XBB variant. Given that 2D4 V_H_H-IgA neutralized Omicron XBB *in vitro* 2.7 to 3.8X more potently than 1C2 and 2D10, respectively, we next tested if 2D4 could also neutralize this SARS-CoV-2 variant *in vivo*. We employed K18-hACE2 transgenic mice (K18-hACE2), in which human ACE2 is expressed under the control of the epithelial cell cytokeratin-18 (K18) promoter (34). We tested intranasal as a route of mucosal administration to deliver 2D4 V_H_H-IgA because this route drives mucosal immunity by class switching IgG to IgA against SARS-CoV-2 (35, 36). Following a post-exposure treatment model (Fig. 6*A*), we observed that intranasal administration of 2D4 V_H_H-IgA abated viral infection in the lungs of all recipients (Fig. 6*B*). By comparison, viral infection was detected in all five control animals receiving the same dose of irrelevant antibody. Altogether, we determined that a nanobody fused with the Fc region of an IgA could prophylactically and therapeutically neutralize SARS-CoV-2 with emphasis on the Omicron variants.

## Discussion

IgA is more commonly studied for its potential in mucosal vaccines or local treatments for infections (*e.g.*, respiratory) at mucosal surfaces. During early-onset SARS-CoV-2 infection, IgA neutralizing responses supersede IgG at both systemic and mucosal levels (15). Anti-SARS-CoV-2 IgG peaks at mid-to-late stages of infection, and the levels decrease over time, whereas mucosal IgA levels are less affected due to the sustained RBD-specific memory B cells even 6 months post-infection (37). It is noteworthy that mucosal IgA responses are most robust following primary infection rather than post-vaccination, which is IgG-dominant (38, 39, 40). Similarly, breakthrough infections following vaccination result in stronger circulating IgA responses, which not only provide diagnostic potential but are associated with protection from the breakthrough infection (41, 42). To further emphasize the importance and application of IgA, pregnant women either vaccinated or infected with SARS-CoV-2 induce viral neutralizing IgA, not IgG, in human milk with evidence of transplacental transfer to the fetus (43, 44). Collectively, these studies provide support to the concept that designing a therapeutic based on IgA would be beneficial to treat infections, like SARS-CoV-2, at mucosal surfaces.

In this study, our goal was to design an IgA-based therapeutic to broadly neutralize SARS-CoV-2 both *in vitro* and *in vivo*. During the design phase, we investigated current approaches with IgG as a baseline and moved forward with Fc-fusion proteins because they (1) extend the half-life of the conjugated protein, (2) enhance immunogenicity by increasing uptake *via* Fc-receptors on antigen-presenting cells, and (3) effectively neutralize and reduce injury in several systemic and mucosal infection models (45, 46, 47, 48, 49, 50), including SARS-CoV-2 (51, 52). These referenced fusion proteins incorporated a fragment or domain of the antigen, for instance, the SARS-CoV-2 S1 subunit with the Fc region (CH1-) of IgG. There are only a few reports that describe the generation of IgA-Fc-fusion proteins, and they targeted either Enterohemorrhagic *Escherichia coli* (33, 53, 54) or the ancestral strain of SARS-CoV-2 (27). Instead of an antigen fragment, the IgA-Fc was conjugated with a nanobody (V_H_H), which was chosen for its smaller size, high potency, thermostability, and ease of manufacturing. Notably, V_H_H identified from alpaca, llama, shark, and phage immune libraries were characterized with potent neutralizing activity against several SARS-CoV-2 variants by blocking receptor interaction (55, 56, 57, 58, 59, 60). Moreover, nanobody cocktails have been demonstrated to have synergistic potential against emerging SARS-CoV-2 variants (61, 62). However, V_H_H alone tend to have significantly lower potency due presumably due to monovalent binding and overall avidity (29, unpublished observations). When expressed as an IgG or IgA fusion protein, potency dramatically increases. As such, we designed, produced V_H_H IgA-Fc fusion proteins, and tested mucosal administration as a mode of drug delivery.

To discover high-affinity, functional nanobodies against Delta and Omicron Ba.1 RBD proteins, we immunized llamas and then performed phage display library screening. This approach offers several advantages in comparison to our prior study where we discovered V_H_H1.1 from an open-source naive nanobody library against the spike protein of the ancestral strain (27). Immunized libraries capture the full *in vivo* immune response, including nanobodies that undergo somatic hypermutation as a means of affinity maturation to improve binding to the antigen (63). Naive libraries rely only on their initial diversity, which might not always include high-affinity binders for a given antigen. Moreover, affinity-matured sequences would require fewer rounds of panning to identify strong binders, whereas naïve libraries often require additional *in vitro* maturation, increasing the time and effort required to obtain useful candidates (63). From our two rounds of panning with the immunized phage display library with a size of approximately 5 × 10^8^ transformants, we identified 248 unique nanobody sequences out of 288 positive clones. Our yield is comparable to previous studies that utilized immunized llama libraries to discover RBD-specific V_H_H (58, 64) and significantly better than the low-yield (<60) of positive, unique clones found from naïve llama (65, 66), humanized (67), and synthetic (68, 69) nanobody phage libraries against SARS-CoV-2. Of note, the recent construction of a nurse shark V_NAR_ phage library identified >300 positive clones against the spike S2 subunit (70), which is a much better output than the previously mentioned naïve libraries. However, only 53 of the 327 binders had unique sequences, signifying room for improvement. Besides further exploration of the V_NAR_ phage library, the recent development of a “LamaMouse,” a transgenic model where the llama immunoglobulin heavy chain (IgH) locus is engineered into IgH-deficient mice (71), opens a new platform to discover high-affinity, functional nanobodies to target SARS-CoV-2 and other pathogens.

Out of the 248 unique V_H_H sequences identified herein, the top fifty candidates with the highest auto- and cross-reactivity toward the RBD proteins were cloned and expressed as IgA-Fc fusion proteins. Two rounds of down selection *via* ELISA and pseudovirus neutralization assay (IC_50_ < 40 pM) led us to select the leading three candidates known as 2D4, 1C2, and 2D10. When performing infection with authentic SARS-CoV-2 *in vitro*, we observed 2D10 to have the most consistent, potent IC_50_ values amongst all Omicron VOCs. However, this candidate was not moved forward for testing therapeutic efficacy in an *in vivo* infection model as expression of soluble protein was poor making it not suitable for manufacturing. Sequence alignment of all three candidates demonstrated that 2D10 is the most unique, with only 66.9% and 65.4% similarity to 2D4 and 1C2, respectively. Comparatively, 2D4 and 1C2 have 95.1% similarity with the main amino acid differences located in the CDR3 region. In 2D10, the high number of glutamine residues and hydrophobic residues could potentially cause protein aggregation and toxicity (72) This instability is predicted when analyzed using ProtParam tool on Expasy (73) solely based on sequence, where a score below 40 is considered stable, 2D10 scored 47, while 2D4 and 1C2 both scored around 30. For 2D10 to be a viable therapeutic, its sequence would require reengineering to retain its neutralization efficacy and simultaneously improve its protein solubility and expression yield stability. For the *in vivo* tests, the main SARS-CoV-2 VOC at the time was the XBB variant, which narrowed our leading candidate to 2D4, given that it neutralized Omicron XBB *in vitro* 2.7X more potently than 1C2. We observed that XBB-infected mice treated with 2D4 V_H_H-IgA had no measurable levels of viral RNA in their lungs, signifying protection from infection. It would be beneficial for subsequent studies to assess other inhalable modes, including aerosolization approaches like nebulizers and dry powder, of mucosal administration for V_H_H-IgA proteins. Success has already been reported for nanobodies and bispecific antibodies using these methods (27, 74, 75, 76), so it stands to reason that V_H_H-IgA proteins are applicable as well. It would also be relevant in the future to design the V_H_H-IgA proteins as multi-specific, multi-affinity antibodies (*e.g.*, bispecific) in either dimeric or tetrameric secretory forms of IgA, where the increased valency may further elevate their therapeutic efficacy as seen with other monomeric nanobodies (77, 78, 79, 80, 81, 82, 83). Of note, one study in Syrian hamsters has shown that dimeric IgA may have a paradoxical outcome in promoting SARS-CoV-2 nasal infection and injury while reducing lung infection (84). This warrants further experimentation.

Overall, our study demonstrates the continued promise in utilizing nanobodies as an effective therapeutic and the opportunity to expand Fc-fusion therapeutics to consider an IgA blueprint during drug development, predominantly in the context of mucosal-related diseases. The findings presented herein provide a steppingstone for further research and development in this area, including the optimization of drug design and delivery of IgA-based therapeutics.

## Experimental procedures

### Antigen and V_H_H cloning, expression, and purification

The RBD sequence (a.a. 319–541) from SARS-CoV-2 spike glycoprotein for the ancestral strain, Alpha, Beta, Lambda, Gamma, Kappa, Delta, Mu, Omicron Ba.1, and Omicron Ba.four-fifths variants were cloned into pcDNA3.1 in-frame with Osteo signal peptide (MRAWIFFLLCLAGRALA) on the N-terminal and 6X His tag on the C-terminal as previously described (17, 27). Nanobody (V_H_H) sequences were identified and cloned into the pcDNA3.1-Osteo-His vector in-frame with human IgA-Fc to generate V_H_H-IgA fusion proteins as previously described (27). All constructs were transiently transfected into Expi293 cells and purified by immobilized metal chelate affinity chromatography using nickel-nitrilotriacetic acid (HisPur Ni-NTA Resin) (17, 27) Any cell lines used were obtained from vendors or ATCC, which verified the authenticity of the cells that were sent. From those vials, a master cell bank was generated from which vials were thawed for use.

### Llama immunization and phage display library

Immunizations of llamas and library constructions were performed as previously described (29). Two male llamas underwent a 6-week immunization regimen with an immunogen consisting of a combination of Delta (B,1617.2) and Omicron Ba.1 (*alias* B.1.1.529) RBD. Delta RBD was produced by cloning amino acids 319 to 541 into pcDNA3.1 in-frame with Osteo signal peptide (MRAWIFFLLCLAGRALA) on the N-terminal and 6X His tag on the C-terminal preceded by a thrombin cleavage site. Prior to use for immunization, the His tag was cleaved using the Thrombin CleanCleave Kit (Millipore Sigma) following the manufacturer’s instructions and purity determined by SDS-PAGE (Fig. 1*A*). As part of the quality control, it was confirmed that there was no change in immunoreactivity of the cleaved RBD as compared to the recombinant protein with a His-tag (Fig. S1). Omicron RBD (B.1.1.529, AA319–537), expressed from HEK293 cells, was obtained from Acro Biosystems (SPD-C522e). One week post final injection, RNA was isolated from peripheral blood lymphocytes, and library constructions were performed by amplifying nanobody (V_H_H) genes and ligating them into a phagemid vector. Two phage display libraries were generated and panned against immobilized Omicron Ba.1 or Delta RBD, and output from those were used for subsequent auto- and cross-panning (Omicron-Omicron, Omicron-Delta, Delta-Delta, Delta-Omicron). Reactive phages were eluted and used to infect exponentially growing *E. coli* TG1 cells. Periplasmic extracts were prepared according to standard protocols involving overnight production in 2 YT medium and induction with IPTG. Extracts were tested for binding Omicron Ba.1 and Delta RBD antigens *via* ELISA using HRP conjugated anti-Camelid VHH antibody cocktail (GenScript A02016). Based on O.D.450 nm readings from ELISA and non-repetitive amino acid V_H_H sequences, unique candidate genes were selected for further characterization.

### Enzyme-linked immunosorbent assay

Purified V_H_H-IgA proteins were tested on ELISA for reactivity against recombinant RBD proteins (17). Briefly, 96-well plates were coated with RBD proteins at 2 μg/ml overnight at 4 °C, followed by blocking with 1% BSA with 0.05% Tween-20 in PBS. Purified V_H_H-IgA diluted in blocking buffer was added and incubated for 45 min at room temperature. After washing, plates were incubated with horseradish peroxidase-conjugated goat-anti-human IgA (1:30,000, Southern Biotech) for 45 min at room temperature. The signal was developed using 3, 3′, 5, 5′-tetramethylbenzidine reagents (Two components TMB; SeraCare). Absorbance at an optical density of 450 nm (O.D. 450) was measured on an Epoch precision plate reader (BioTek) using Gen5 software.

### Pseudotyped virus neutralization assay

The production of pseudotyped SARS-CoV-2 and the neutralization assay of nanobody fusion proteins were performed as previously described (17, 27). In brief, a pseudovirus was generated by second-generation lentiviral packaging plasmids (psPAX2: Addgene Plasmid #12260 and pLenti-Luc: Addgene Plasmid #17477), which contain a luciferase gene to direct luciferase expression in target cells (85). A human ACE2 (HEK293T) stable cell line (Creative Diagnostics # CSC-ACE01) was used for pseudovirus infection with puromycin as the selection agent. The appropriate volume of pseudovirus was pre-incubated with serially diluted V_H_H-IgA for 1 h at room temperature before being added to 293T cells expressing hACE2. The infection was quantified 48 h post virus induction by luciferase detection with Bright Glo luciferase assay (Promega) and read in a VICTOR Nivo plate reader (PerkinElmer) for light production.

### Biosafety

All study protocols were reviewed and approved by Environmental Health and Safety and the Institutional Review Board at the University of Massachusetts Chan Medical School prior to study initiation. All experiments with SARS-CoV-2 were performed in a biosafety level 3 laboratory by personnel equipped with powered air-purifying respirators.

### Infection with authentic SARS-CoV-2 *in vitro*

Vero E6 cells were pretreated with various concentrations of V_H_H-IgA for 2 h before being infected with SARS-CoV-2 Omicron VOCs at MOI = 0.05. After 24 h of infection, the cells were collected with Trizol (Invitrogen). Total RNA was extracted with the Direct-Zol RNA miniprep kit (Zymo) per the manufacturer’s instructions, and equal amounts of RNA were reverse-transcribed using the iScript cDNA Synthesis Kit (Bio-Rad). Diluted cDNAs (1:100 final) were subjected to qPCR analysis using iQ SYBR Green Supermix Reagent (Bio-Rad).

### SARS-CoV-2 infection in mice

All animal experiments were approved by the Institutional Animal Care and Use Committee at the University of Massachusetts Chan Medical School. Animals were kept in a specific pathogen-free environment. Hemizygous K18-hACE2 C57BL/6J mice (strain: 2B6.Cg-Tg (K18-ACE2)2Prlmn/J) were obtained from The Jackson Laboratory (86). Animals were housed in groups and fed standard chow diets. Sample sizes used are in line with other similar published studies (79). For the animal protection experiment, eight to 12-week-old male mice were anaesthetized with intraperitoneal injection of ketamine (100 mg/kg body weight) and xylazine (10 mg/kg body weight). Mice were then infected intranasally with 5.86 × 10^5^ PFU of the SARS-CoV-2 VOC XBB. Nanobodies were intranasally delivered at 6 h post-viral infection at a 10 mg/kg dose. For viral load assessment, mice were euthanized 72 h post-infection using isoflurane. Lung tissue was collected, homogenized, and then RNA was extracted using the Direct-Zol RNA miniprep kit (Zymo), following the manufacturer's protocol. RNA was reverse-transcribed using the iScript cDNA synthesis kit (Bio-Rad) and SARS-CoV-2 RNA levels *via* qPCR.

### SARS-CoV-2 RNA analysis *via* qPCR

A 5 ng sample of cDNA from both *in vitro* and *in vivo* live virus assays was analyzed by qPCR using iQ SYBR Green Supermix (Bio-Rad). Gene expression was normalized to GAPDH or TATA-binding protein housekeeping genes. Relative mRNA expression was calculated using the change in cycling threshold (ΔΔCt) method as 2^∧^−ΔΔCt. Specificity of the RT-qPCR amplification was confirmed through melting curve analysis. The sequences of primers used in this study were: SARS-CoV-2-N F CTCTTGTAGATCTGTTCTCTAAACGAAC, R GGTCCACCAAACGTAATGCG.

### Structural modeling

The amino acid sequences for the leading V_H_H-IgA candidates (2D4, 1C2, 2D10) and the ancestral strain and RBD variants (Delta, and Omicron Ba.1) were used as a single FASTA input. The structural models for the nanobody-RBD complex were predicted using AlphaFold-Multimer (87) version 2.3.2 on a high-performance computing SCI cluster with NVIDIA A100 GPUs to generate 20 models per nanobody-RBD pair. The top five ranked predicted models are shown in Fig. S3. Using PyMol version 2.0.7, the highest confidence predictions (confirmed by per-residue confidence as measured by pLDDT) (Fig. S4) for each nanobody-RBD pair were aligned on the RBD for visual inspection. The predicted aligned error matrices were additionally inspected to assess the confidence in the relative position and orientation of the binding mode. The structural images were generated using PyMol version 2.0.7.

### Statistical analysis

Statistical calculations were performed using Prism version 9.0 (GraphPad Software). EC_50_ and IC_50_ values were calculated by sigmoidal curve fitting using nonlinear regression analysis. For comparisons of two groups, two-tailed Student’s *t* tests were performed. Multiple comparison analysis was performed using one-way ANOVA.

## Data availability

The data generated in this study are available upon request from the corresponding author.

All structural models generated using AlphaFold2 of Spike RBD variants bound to leading nanobodies are available in the ModelArchive entry ma-rbdnb with the accession codes ma-rbdnb-01 through ma-rbdnb-09.

## Supporting information

This article contains supporting information.

## Conflict of interest

The authors declare that they have no conflicts of interest with the contents of this article.
