# Supplementary material for: Engineered IgA-Fc fusion protein with bioactive nanobody neutralizes SARS-CoV-2 variants with mucosal delivery potential
Source: J Biol Chem. 2026 Jan 28;302(3):111210. doi: 10.1016/j.jbc.2026.111210 (PMC13080570; doi:10.1016/j.jbc.2026.111210)
Supplement: Supporting Information [file mmc1.docx]

**Supporting Information**

**Engineered IgA-Fc fusion protein with bioactive nanobody neutralizes SARS-CoV-2 variants with mucosal delivery potential**

**Authors:** Rachel M. Golonka^1^, Lauren E. Intravaia^2^, Ala M. Shaqra^2^, Qi Li^1^, Yongzhi Chen^1^, Fiachra Humphries^1^, Nese Kurt-Yilmaz^2^, Kate A. Fitzgerald^1^, Celia A. Schiffer^2^, Yang Wang^1^, Lisa A. Cavacini^1,^*

Affiliations:

^1^Division of Innate Immunity, Department of Medicine, University of Massachusetts Chan Medical School, Worcester, MA 01605

^2^Department of Biochemistry and Molecular Biotechnology, University of Massachusetts Chan Medical School, Worcester, MA 01605

Current Address:

Rachel Golonka, Nona Biosciences, Natick, MA 01760

Qi Li, Novartis, Cambridge MA 02139

Yang Wang, AMB Biomed Consulting, Worcester, MA; Timberlyne Therapeutics, San Diego, CA 92122

***Corresponding author**

Lisa A. Cavacini, PhD

Professor, Department of Medicine

University of Massachusetts Chan Medical School

Worcester, MA 01605 USA

Phone: 508-856-5748

Email: lisa.cavacini@umassmed.edu


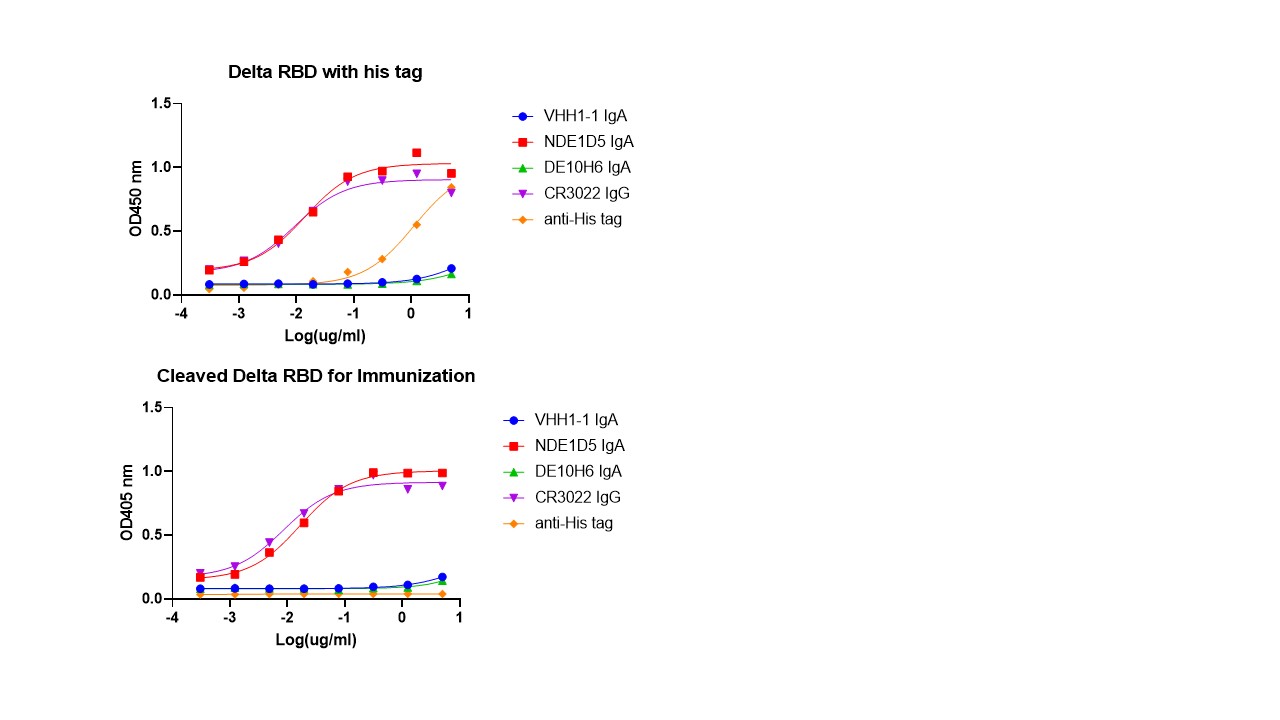


**Figure S1**: Immunoreactivity of anti-RBD and anti-His antibodies with Delta RBD. Delta RBD with a thrombin cleavage site proximal to a 6-His tag was cleaved to remove the tag prior to use for immunization. Plates were coated with Delta RBD prior to cleavage and post removal of the His-tag. Known monoclonal antibodies to RBD or to the his-tag were added and detected using alkaline phosphatase conjugated secondary antibody followed by pNPP substrate. Optical density was determined at 405nm and plotted as a function of antibody concentration.


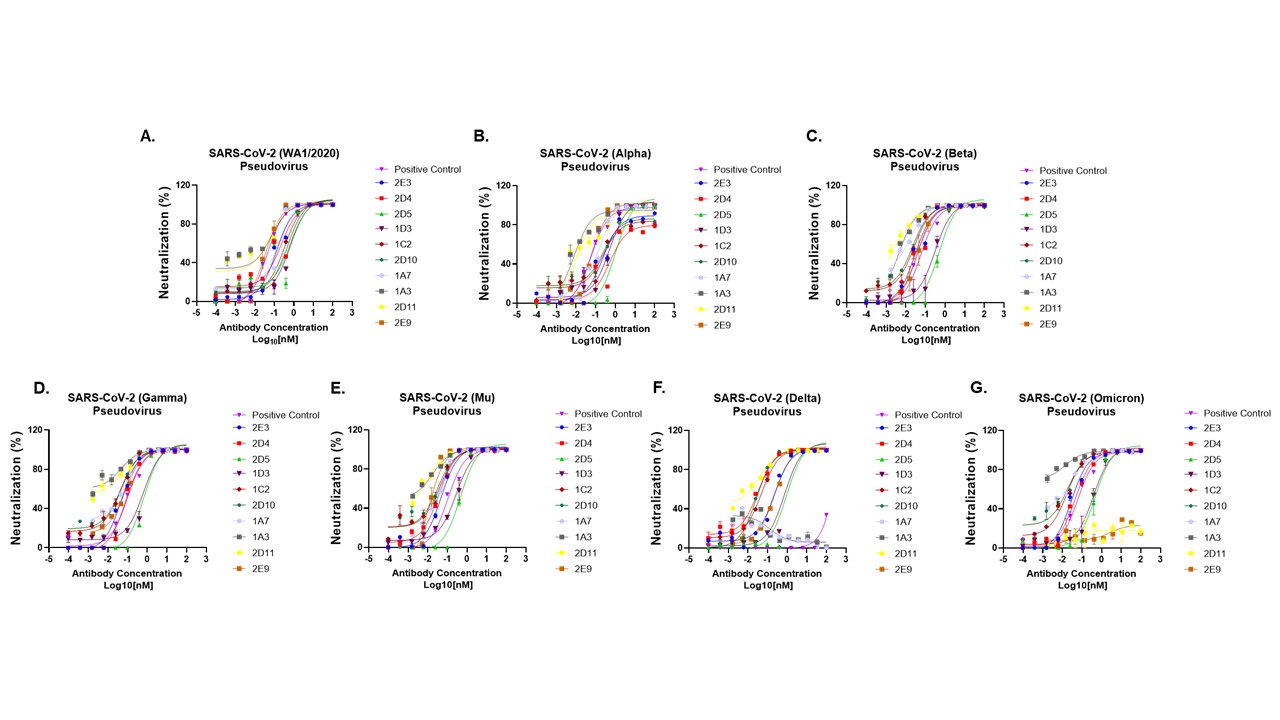


**Figure S2**: Top 10 anti-RBD V_H_H-IgA candidates against 7 pseudotype virus strains of SARS-CoV-2. These are the individual neutralization curves for each V_H_H-IgA against the seven strains as summarized in Figure 3.

>2D4

EVQLVESGGGLVMPGDSLTLSCAASGRAISDYGMGWFRQAPGKPRVWVATISRDSGATLYSDSVRGRFTISRDNDKNTVSLRMKFVKLEDTALYFCGAADYRQWYSPAAYQFWGQGTQVTVSS

>1C2

EVQLVESGGGLVMPGDSLTLSCAASGRAISDYGMGWFRQVPGKQRVWVATISRDSGATLYSDSVRGRFTISRDNDKNTVSLRMKFVKLEDTALYFCGAADYRSWYSPGAYHDWGQGTQVTVSS

>2D10

EVQLVESGGGSVQTGDSLRLACAASGRTFSGTSMGWFRQPAGRERQFVGGITWNDGTRFYAPSVKGRFTISRDIDKMTMSLQMNNLKPEDTAVYYCTPAQKGVGTQASTYDYCGLGTHFTDSS

**Table S1**: Amino acid sequence of lead V_H_H.


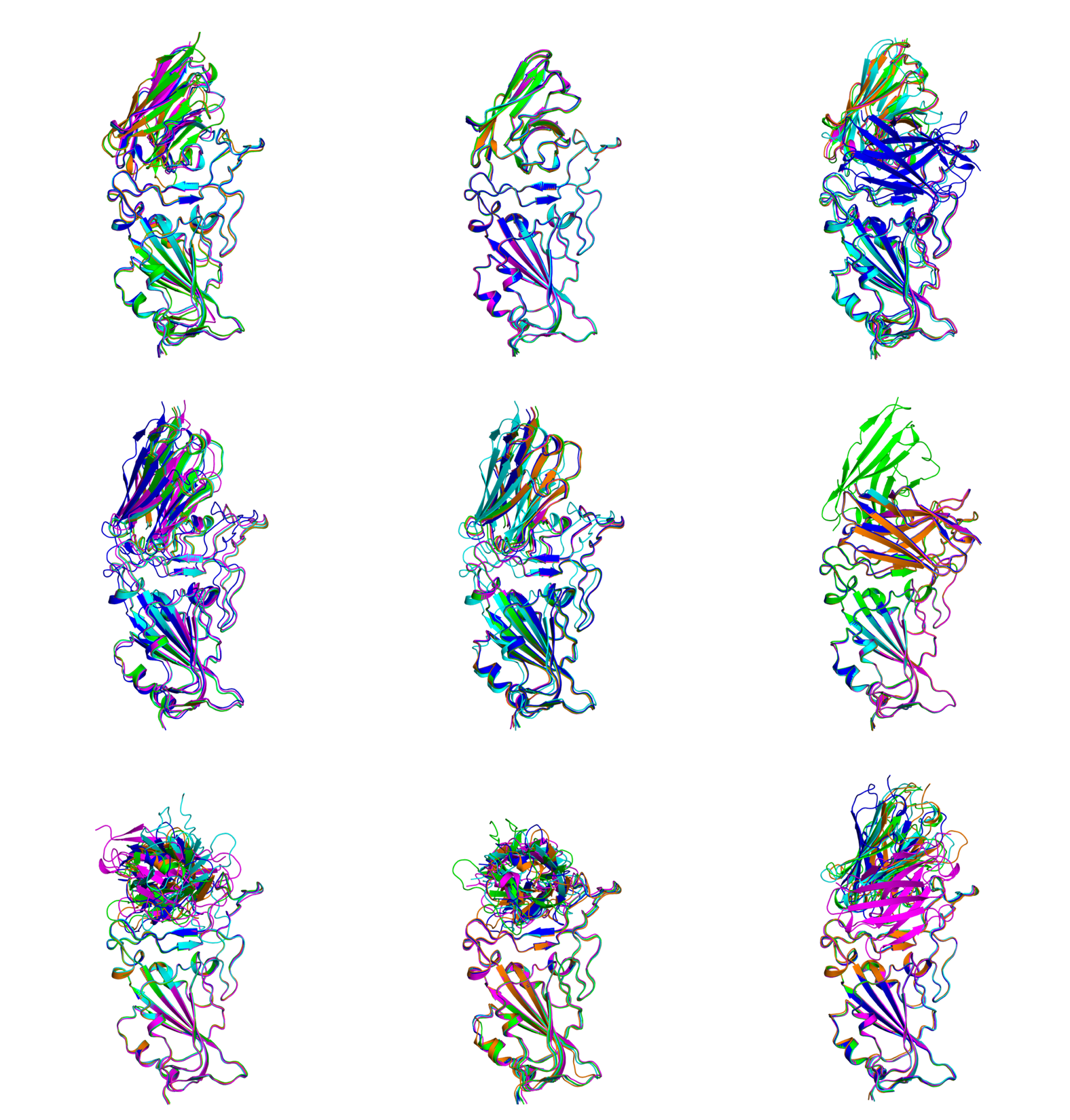


1C2

1C2

1C2

WA

WA

WA

Delta

Delta

Delta

Omicron

Omicron

Omicron

2D4

2D4

2D4

2D10

2D10

2D10

**Figure S3:** Top 5 predicted structures of Spike RBD variants bound to leading nanobodies from AlphaFold2. All models were aligned on the Spike RBD to highlight the different nanobody binding conformations predicted. The predicted structures are colored according to ranking: the most confident prediction is blue, second is cyan, third is magenta, fourth is orange, and fifth is green.


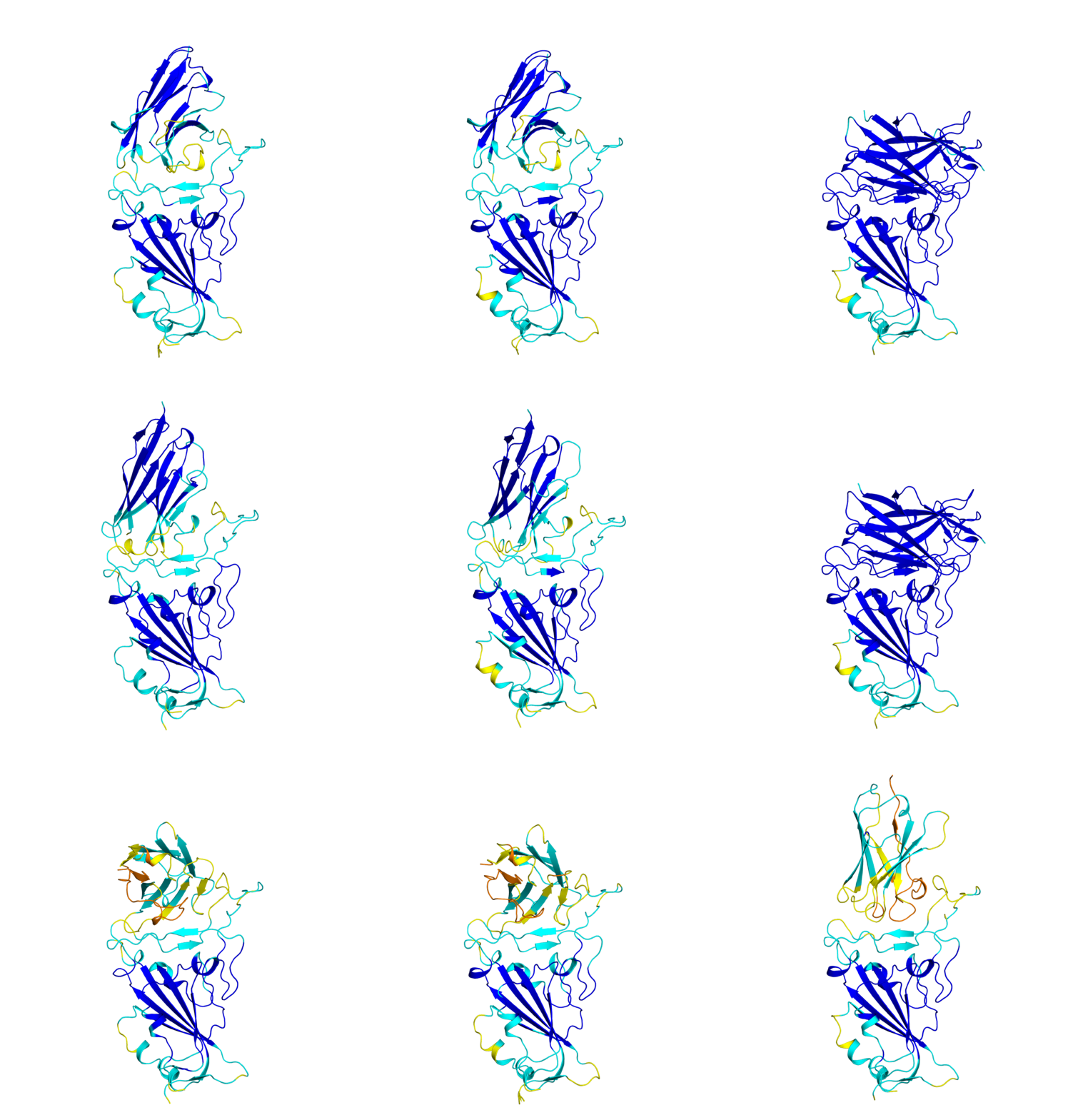


plDDT > 90

90 > plDDT > 70

70 > plDDT > 50

plDDT < 50

1C2

1C2

1C2

WA

WA

WA

Delta

Delta

Delta

Omicron

Omicron

Omicron

2D4

2D4

2D4

2D10

2D10

2D10

**Figure S4:** Highest confidence prediction for each Spike RBD variant-nanobody complex colored according to the AlphaFold2 plDDT values.
